# Evaluation of Artificial Intelligence (AI) Scribes in Medical Practice: Cross-Regional Analysis

**DOI:** 10.1192/bjo.2025.10184

**Published:** 2025-06-20

**Authors:** Anju Soni, Ian Treasaden

**Affiliations:** ^1^Canberra Health Services, Canberra, Australia; ^2^West London NHS Trust, London, United Kingdom

## Abstract

**Aims:** This study aimed to evaluate the implementation of AI scribes in medical practice across Australia and England, focusing on assessing current adoption rates, measuring awareness of perceived benefits and potential risks and developing recommendations for safe and effective deployment.

**Methods:** A comprehensive survey was conducted across 50 medical practitioners equally distributed between Australia and England. Practitioners were from both urban (60%) and rural (40%) environments, with a representation across experience levels: junior (30%), mid-career (45%), and senior (25%) practitioners. Data was collected over 4 weeks through a semi-structured questionnaire.

**Results:** The analysis revealed significant insights into AI scribe adoption and perception across both countries. Of the surveyed practitioners, 28% (14) are currently utilising AI scribes in their practice, while nearly half (48%) are actively considering implementation. The remaining 24% expressed no immediate plans to adopt this technology. Reported AI scribe benefits were notably high among respondents, with time-saving potential being the most recognised advantage (90% awareness). Practitioners demonstrated strong recognition of the technology’s ability to reduce administrative burden (84%) and improve patient interaction (76%). However, the assessment of documentation quality improvements was lower at 62%.

Risk awareness varied significantly across different aspects. Privacy concerns dominated the risk perception landscape, with 78% of practitioners expressing awareness of potential privacy issues. Clinical accuracy risks and legal liability concerns were acknowledged by 70% and 64% of responders respectively. A crucial finding was that only 42% of practitioners were aware of their medical defence union’s position on AI scribe usage, revealing a significant knowledge gap in professional liability coverage.

Among current users, satisfaction levels showed a mixed picture. While 64% reported positive experiences (21% very satisfied, 43% somewhat satisfied), a notable portion remained neutral (22%) or expressed dissatisfaction (14%). Implementation concerns centred primarily around training requirements (80%) and system integration challenges (72%), with medical defence coverage emerging as a significant concern (62%).

**Conclusion:** The study highlights the critical need for healthcare providers to establish comprehensive implementation strategies that address both technical and legal considerations. Practitioners in both regions must prioritise verification of medical defence union coverage before adopting AI scribes. UK medical defence unions have clearer guidelines compared with Australian medical defence organisations. Australian practitioners should align their implementation with RACGP digital health guidelines, while UK practitioners need to ensure NHS Digital compliance.

The findings emphasize that successful AI scribe implementation requires a balanced approach that addresses technical integration, risk management, and insurance coverage. The high level of interest, coupled with significant uncertainty about medical defence coverage, indicates a clear need for professional organisations to provide more detailed guidance on this emerging technology.

